# URTICARIA

**DOI:** 10.4103/0019-5154.55638

**Published:** 2009

**Authors:** Sanjay Ghosh

**Affiliations:** *From the Urticaria Clinic, Institute of Allergic and Immunological Skin Diseases, Kolkata, India.*

When the editor of the Indian Journal of Dermatology (IJD) approached me with a request to be the editor of a symposium on ‘uriticaria’, to be published in this esteemed journal, I felt delighted and grateful to him for rightly selecting an ever-growing, contemporary, and challenging subject. Entrusted with this daunting task, I decided to directly approach the most legendary persons on this subject from different parts of the globe, namely, Drs. H Maibach, C L Goh, and A K Bajaj from the United States, Singapore, and India, respectively. All of them responded eagerly to my request and kept their promise by sending their articles in time, in spite of their extremely hectic schedules. On behalf of the IJD, I must express our sincere thanks and gratitude to all of them.

The symposium comprises four articles on as many important aspects of this important condition: contact urticaria, chronic autoimmune urticaria, management of difficult difficult urticaria, and the updates on the disease.

Keeping in mind his monumental contribution to the world of dermatology, we are dedicating this symposium on ‘Urticaria’ in IJD to Prof. H Maibach as a tribute to him on behalf of all the dermatologists in India.

In conclusion, I must congratulate the editor, the executive editors, the associate editors and the whole team of IJD for publishing such a symposium on urticaria.

